# Health Related Quality of Life of Stroke Survivors: Experience of a Stroke Unit

**Published:** 2012-09

**Authors:** S. A. Abubakar, S. A. Isezuo

**Affiliations:** 1 *Department of medicine Ahmadu Bello University Teaching Hospital, Zaria, Nigeria;*; 2 *Department of medicine Usmanu Danfodiyio University Teaching Hospital, Sokoto, Nigeria*

**Keywords:** health related quality of life, stroke survivors, SIS-16, Sokoto

## Abstract

**Background:**

Stroke has a major impact on survivors including Health related Quality of life (HRQoL). HRQoL measurements are potentially more relevant to patients than measurements of impairments or disability and are an important index of outcome after stroke that can facilitate a broader description of disease and outcome. This study examined the factors associated with HRQoL of stroke survivors.

**Methods:**

In a cross-sectional and descriptive correlational design, 62 patients were prospectively enrolled and interviewed 3 months post stroke in neurology out-patient clinic. After case identification, functional status (handicap) was determined using the Modified Rankin Scale (MRS), while Zung Depression Self-Rating Scale (ZDS) was used to determine presence of depression. HRQoL was assessed using the Stroke Impact Scale-16 (SIS-16). Age, sex, duration of formal education, depression and degree of disability were correlated with HRQoL in multiple logistic regressions.

**Results:**

The mean age of patients was 54.4 ± 9.9 years. Mean duration of formal education was significantly higher in males than females (*p* value=0.007). About one third (29%) of the stroke survivors were depressed and more than half (54.8%) had good recovery. Function status measured by modified Rankin Scale and depression were independent determinants of poor HRQoL.

**Conclusion:**

Functional status and depression were identified as independent factors affecting HRQoL of stroke survivors.

## INTRODUCTION

Stroke is the world’s third leading cause of death and the most important cause of severe adult disability (1). Stroke is often catastrophic and affects all aspects of an individual’s life (2), and unlike other disabling conditions the onset of stroke is sudden leaving the individual and family ill-prepared to deal with the sequelae (2). The long term consequences of stroke have been recognized. Epidemiological studies of stroke in North-western Nigeria have focused on mortality and risk factors profile but not on quality of life issues (3, 4). Quality of life related to stroke and life satisfaction after stroke is important health care issues that have not received sufficient attention in sub-Saharan Africa (5).

Stroke causes sufficient decrease in quality of life even among those who have no post stroke disability (6). In other populations multiple risk factors including age (7, 8), gender (9), dependency in activities of daily living ADL/disability (8), social support (10), depression (8, 9, 11), institutionalization (8) and diabetes (7, 8) have been associated with poorer HRQoL in stroke survivors. Results from these studies were inconclusive in part because of the variability in HRQoL measurements (SF-36 (9, 12), Sickness Impact Profile (9), Stroke Impact Scale (6) and many others (10)). The Short Form-36 (SF-36) has been used in many clinical trials to asses HRQoL in stroke survivors, but ceiling and floor effects have limited the ability of these instrument to evaluate these stroke patients’ disability or Health outcome over time (13, 14) i.e large portion of patients are located in the highest or lowest score of the instrument reducing the instrument’s ability to detect changes. To address these limitations, the Stroke Impact Scale (SIS), a stroke specific outcome measure, and more comprehensive measure of Heath Outcome was designed. This SIS version 3.0 includes 59 items and assesses 8 domains (strength, hand function, ADL/instrumental ADL, mobility, communication, emotion, thinking and social participation). Sixteen items from 4 of the 8 domains can be combined into an overall physical component score. This composite domain of physical function includes16-items and is referred to as the SIS-16 (14, 16). The aim of present study is to determine factors that affect Health Related Quality of Life (HRQoL) of stroke survivors 3 months after stroke. The results may provide valuable information about strategies that professionals and provider of stroke care can address to improve HRQoL.

## SUBJECT/METHODS

The study was carried out between March 2009 and June 2010 on patients managed by the Neurology Unit of Usmanu Danfodiyio University Teaching hospital (UDUTH) Sokoto with diagnosis of stroke. Consecutively presenting and consenting stroke patients seen at the neurology clinic were prospectively enrolled. Included in the study are stroke patients who had survived up to 3 months post stroke. Brain CT-Scan was used for clinical definition of stroke, however where brain CT-Scan was not available, as is the practise in most low-income countries diagnosis of stroke was based on WHO clinical definition (17). The clinical distinction of stroke from other disorders has a sensitivity of 95% and specificity of 97%. Excluded from the study are those who had stroke for less than three months and those who could not communicate fluently nor had close, suitable and reliable proxies.

### Methods

After case identification and verification, demographic data including age, gender, and duration of formal education and co morbidities were obtained from patients and medical records using structured questionnaire. Disability/handicapped (3-month post stroke) were determined using Modified Rankin Scale (MRS) (18). MRS is commonly used to assess disability after stroke. The MRS attempts to measure functional independence, incorporating the WHO components of body function, activity and participation. The disability categories are ordered from 0 to 5, with 0 representing no symptoms at all while 5 represent severe disability (18). Depression was determined using Zung depression self-rating Scale (ZDS) (19). The Zung Self-Rating Depression Scale is a 20 item self-report questionnaire that is widely used as a screening tool, covering affective, psychological and somatic symptoms associated with depression. The questionnaire takes about 10 minutes to complete, and items are framed in terms of positive and negative statements. It can be effectively used in a variety of settings, including primary care, psychiatric, drug trials and various research situations. Each item is scored on a Likert scale ranging from 1 to 4. A total score is derived by summing the individual item scores, and ranges from 20 to 80. Most people with depression score between 50 and 69, while a score of 70 and above indicates severe depression (20).

Health related Quality of life (HRQoL) of stroke survivors was measured 3 months after stroke by a face to face interview using Stroke Impact Scale-16 (SIS-16) (6). SIS-16 assesses physical function. The respondent answered with the text associated with the number (not difficult at all=5 or could not do at all=1) for an individual question. The four domain were aggregated to create one physical domain (as previously explained) and were transferred to a scale with total score ranging from 0 to 100 with 0 been the worst possible score and 100 the best possible score. As such the floor effect is defined as score of 0 indicating that the patients are unable to perform all physical functioning and ceiling effect is a score of 100, indicating patients are able to perform all physical activities. A good score was defined as a score of ≥75 points which is equivalent to independent function or MRS of 2 or less (6).

Hypertension was taken as positive history, use of anti-hypertensive drugs or persistently elevated blood pressure (>140/90 mmHg) while on admission. Diabetes mellitus was regarded as a positive history, use of hypoglycemic agents or a fasting plasma glucose of >7.0 mmol/L on two occasions. Stroke was defined as a clinical syndrome of sudden onset of rapidly developing symptoms and signs of focal or global cerebral deficit with symptom lasting more than 24 hours or leading to death with no apparent cause other than vascular origin (21).

Data were analyzed using SPSS 15.0, means and standard deviations were generated. Non-continuous variables were compared using frequencies or percentages. Multiple (multivariate) logistic regression was used to determine the effect of independent variables on stroke impact scale (SIS-16). P value less than or equal to 0.05 were considered statistically significant.

## RESULTS

Seventy two stroke survivors were seen during the study period, of which 7 did not consent to participate in the study while 3 were globally aphasic and could not communicate and were thus excluded. The mean age of the stroke survivors was 54.4 ± 9.9 years comprising 33 males and 29 females as shown in Table 1. Systemic hypertension was the most common co morbid condition occurring in 62.9% while 6.7% had history of previous TIA/stroke. 30 (48.39%) had brain CT-Scan confirmation of stroke, of these cerebral infarction was seen in 18 (60%), primary intracerebral haemorrhage in 9 (30.0%), while subarachnoid haemorrhage was present in 1 (3.3%) of the patients. Two (6.7%) patients had normal brain CT-Scan and were classified as cerebral infarction. 54 (87.1%) presented to the hospital on a weekday and most of the patients 52 (83.9%) resides in urban area.

**Table 1:** Baseline characteristic of stroke patients

Characteristics of stroke patients	Value

Gender: male/female	33/29
Age in years: mean (± SD)	54.4 (9.9)
Duration of formal education in years: mean (± SD)	6.7 (6.0)
Co morbidities; N (%):	
Hypertension	39 (62.9)
Diabetes mellitus	5 (8.1)
Cigarette smoking	6 (9.7)
Alcohol	2 (3.2)
Previous stroke	4 (6.7)
3-month SIS-16: mean (± SD)	68.9 (26.1)
3- month ZDS: mean (± SD)	39.2 (13.6)
Admission NIHSS: mean (± SD)	7.3 (3.2)

SIS, stroke impact scale; ZDS, Zung depression self-rating scale; SD, standard deviation; N, number; NIHSS, National Institute of Health Stroke Score.

Admission systolic and diastolic blood pressure of the stroke survivors were 164.3 (32.14) and 100.8 (18.5) mmHg respectively and was not statistically different in both males and females (Table 2).

**Table 2:** Clinical characteristic of stroke patients stratified by gender

Characteristics	Total (N=62)	Female (N=29)	Male (N=33)	*P* value

Age in years				
Mean (± SD)	54.4 (9.9)	56.6 (8.9)	52.4 (10.5)	
range	21-75	42-75	21-70	*P*=0.093
3-month SIS-16				
mean (± SD)	68.9 (26.1)	66.8 (23.7)	70.8 (13.8)	
range	23-100	30-98	23-100	*P*=0.55
3-month ZDS				
mean (± SD)	39.2 (13.6)	40.0 (14.5)	38.5 (12.9)	
range	20-68	20-68	20-60	*P*=0.53
SIS-16 category; N (%)				
good outcome	34 (54.8)	16 (55.2)	18 (54.5)	*P*=0.96
poor outcome	28 (45.2)	13 (44.8)	15 (45.5)	* *
ZDS category; N (%)				
depression	18 (29)	8 (27.6)	10 (30.3)	*P*=0.81
normal	44 (71)	21 (72.4)	23 (69.7)	* *
Duration of formal education in years				
mean (± SD)	60.7 (6.0)	4.6 (5.6)	8.6 (5.8)	
range	0-16	0-16	0-16	*P*=0.007
3-months MRS				
mean (± SD)	1.9(1.1)	1.8(1.0)	2.0(1.2)	*P*=0.48

Duration of formal education was significantly higher in males than female.

SIS-16 was not statistically different between males and females but females have significantly lower educational attainment than males. 29% of stroke survivors were depressed at time of interview as shown in Table 2. In a multivariate logistic regression, degree of handicap measured by Modified Rankin Scale and presence of depression measured by zung depression Self-Rating Scale were independent determinants of poor HRQoL in stroke survivors (Table 3).

**Table 3:** Determinants of health related quality of life using multivariate logistic regression

Variable	Odd ratio	95% CI	Coefficient	*P* value

3-month MRS	2.42	1.21-4.85	0.89	0.012
3-months ZDS	1.08	1.01-1.15	0.08	0.011
Admission DBP	0.97	0.90-1.04	-0.03	0.397
Admission SBP	1.01	0.97-1.05	0.013	0.513
Age in years	0.97	0.90-1.05	-0.029	0.488
Duration of FE in years	0.86	0.73-1.01	-0.151	0.068
Sex (male/female)	2.25	0.45-11.23	0.813	0.32

MRS, modified rankin scale; DBP, diastolic blood pressure; SBP, systolic blood pressure; FE, formal education; ZDS, Zung depression self-rating scale.

## DISCUSSION

This cross-section of stroke survivors were predominately middle-aged (54.4 years) population. This is similar to the age population that has been described at the same study site (3) and region (4), but a decade below the average age of stroke in other populations (13, 22). The effect of age on HRQoL in stroke survivors in the literature has remained inconclusive. While some found that age had no negative influence on quality of life of stroke survivors (13, 23) a finding that is similar to ours, others found that age had powerful influence on quality of life of stroke survivors (6, 24). The average duration of formal education was significantly higher in males, this could probably be explained by cultural and religious reason in the region as the women tends to remain at home to look after the family. The level of educational attainment also had no influence on quality of life of stroke survivors. Majority (54.8%) of the survivors had good outcome and satisfactory quality of life and mean MRS was less than 2. The degree of handicap measured by MRS had negative influence on QoL. Patients that remained disabled 3 months after stroke had poor QoL. This is in agreement with findings by Hackett *et al*. (25), but some studies have reported poor QoL even in patients with no disability post stroke (6). Level of handicap was also found to be a determinant of poor HRQoL in multivariate logistic analysis. Less than a third (29%) of stroke survivors was depressed using Zung Depression Self-rating Scale at 3 months which is similar to 19-30% in stroke series by Hacket *et al*. (25). Post stroke depression is a treatable condition and early diagnosis is of paramount importance to prevent progression to chronic depressive disorder, as post-stroke depression have been found to be associated with increased chances of suicidal ideation (26). Depression also slows down the process of rehabilitation and was found to negatively affect HRQoL. The negative effect of post stroke depression has also been reported by Williams *et al*. (27). Gray LJ *et al*. (28) found that in acute ischemic stroke female patients had lower QoL score at 6 months than men especially in domain of physical and mental function, but a recent study that looked at life satisfaction of stroke survivors in Luxemburg and north-eastern Portugal found that life satisfaction was higher among women and lower among subjects with impaired motor function. Quality of life was also directly related to that of the caregivers (29). Our study did not find any relationship between gender and stroke outcome including QoL at 3 months.

Outcome in female patients with stroke has been reported to be worse than in males with an increased risk of dependency and institutionalization. This study showed that even though females have lower educational attainment than males, there was no association between sex and SIS-16 values. This lack of association may be because the females were not significantly older than males in this cohort as opposed to studies where females were much older and likely to be more frail hence the possibility of poorer HRQoL. Admission systolic and diastolic blood pressure was not found to have any association with HRQoL of stroke survivors. This is in disagreement with one study that reported a novel association of elevated admission blood pressure with poor QoL (30). Admission blood pressure has been related to outcome of stroke by Ojini *et al*. (31) where he reported that patients with very high blood pressure and low blood pressure have poor outcome measured by modified Rankin Score.

In conclusion the presence of disability and clinical depression were independent determinant of poor heath related quality of life, this study also highlights the importance of considering HRQoL in assessing stroke outcome.
